# Sesame Immunotherapy: A Systematic Review and Narrative Synthesis

**DOI:** 10.1002/clt2.70206

**Published:** 2026-09-29

**Authors:** Joanna Zielińska, Katarzyna Kunkiel, Anna Krupa‐Łaska, Marek Kulus, Katarzyna Grzela

**Affiliations:** ^1^ Department of Pediatric Pulmonology and Allergology Medical University of Warsaw Warsaw Poland

**Keywords:** allergen immunotherapy, anaphylaxis, food allergy, oral food challenge, sesame allergy

## Abstract

Sesame allergy is associated with severe allergic reactions, low rates of spontaneous resolution, and significant psychosocial burden. Current management relies on strict avoidance and emergency treatment. Allergen‐specific immunotherapy, particularly oral immunotherapy (OIT), has emerged as a potential disease‐modifying approach, but evidence for sesame allergy remains limited. A systematic search was conducted through August 2025. Eligible studies included participants of any age with confirmed sesame allergy treated with allergen immunotherapy. Primary outcomes were desensitization and sustained unresponsiveness assessed by oral food challenge. Secondary outcomes included adverse events, epinephrine use, quality of life, and mortality. Risk of bias was evaluated using Joanna Briggs Institute tools. Nine studies met the inclusion criteria, including one study identified through citation searching. The evidence consisted, primarily small cohort studies and case series. Single‐allergen sesame OIT achieved high desensitization rates (approximately 85%–97%) across heterogeneous protocols and age groups. Sustained unresponsiveness was reported in a limited number of mainly pediatric cohorts. Most adverse events were mild to moderate; severe reactions requiring epinephrine were uncommon, and no deaths were reported. Adjunctive omalizumab improved tolerability in high‐risk adults. Sesame OIT shows promising efficacy with an acceptable safety profile in specialized settings. However, evidence is limited by small sample sizes, heterogeneity, and short follow‐up. Randomized controlled trials are needed to define optimal protocols and long‐term outcomes.

**Trail Registration:**

The protocol for this systematic review was prospectively registered in PROSPERO (International Prospective Register of Systematic Reviews; Registration number: CRD420251087250)

## 1 Introduction

Sesame is one of the world's oldest oilseed plants, cultivated for its rich nutritional profile and its wide culinary use. Sesame is widely used in the food industry owing to its favorable technological properties, such as emulsifying capacity and stability, as well as its rich nutritional profile, including high fat and protein content, minerals, B vitamins, and bioactive compounds including lignans, phytosterols, and saponins [1]. It is a common ingredient in bakery products, cereals, confectionery, sauces, and condiments, and is frequently incorporated into Middle Eastern and plant‐based cuisines. As a result, sesame is often present in processed foods, sometimes in small or hidden amounts, making complete avoidance particularly difficult.

Unfortunately, despite its properties and culinary advantages, sesame is one of the major food allergens. Allergies to seed and nut storage proteins are of particular clinical concern because they are associated with severe reactions and a low likelihood of spontaneous resolution. Sesame allergy is therefore potentially life‐threatening and typically requires lifelong dietary avoidance. Its prevalence varies geographically and is estimated at 0.1%–0.9% worldwide [2].

Sesame allergy is most commonly diagnosed between 6 months and 3.5 years of age, although over 25% of patients report adult‐onset disease [3]. Coexisting food allergies are common, affecting approximately 75% of sesame‐allergic individuals, with frequent cross‐reactivity to peanuts (7.1%–69.6%) and tree nuts (10%–69.6%) [3, 4, 5, 6, 7, 8].

In addition, up to 90% of patients have comorbid atopic conditions, including asthma, atopic dermatitis, and allergic rhinitis, as reported in Israeli, British, and American cohorts [3, 9, 10]. Sesame allergy is generally persistent, with spontaneous resolution occurring in only 18%–32% of cases [5, 11, 12, 13].

Diagnosis of sesame allergy is based on a compatible clinical history supported by skin prick testing (SPT), IgE specific to sesame (sIgE) or its components, and, in selected cases, basophil activation test (BAT) [14]. When results are inconclusive, oral food challenge (OFC) is recommended. Tahini paste is most commonly used for OFC, as this study demonstrated that approximately 60% of patients with a negative sesame seed OFC subsequently reacted to tahini [15].

In a survey by Lemoine et al., nearly 50% of families managing multiple food allergies reported sesame avoidance as the most time‐consuming task, and approximately 25% identified it as the greatest financial, social, and emotional burden [16]. Sesame is a potent allergen: based on 250 OFCs the lowest observed dose eliciting objective symptoms was 2.5 mg of sesame protein (≈ 5 sesame seeds), while ED01 was estimated at 0.8 mg (≈ 1–2 seeds), indicating that highly sensitive individuals may react to very small amounts. [17]. In a retrospective pediatric survey, 20% of children experienced allergic reactions following accidental sesame ingestion [18]. The severity of such accidental reactions is highlighted by data showing that among 360 sesame‐related clinical reactions in 327 individuals, nearly 70% resulted in anaphylaxis, almost half required hospitalization, and over one‐third were treated with epinephrine [4]. Data from the United Kingdom indicate that 17% of patients with sesame allergy have experienced potentially life‐threatening reactions, with 65% of severe reactions occurring on first known exposure [10]. In recognition of its prevalence and severity, sesame was included among the “Big Eight” priority food allergens by the FAO/WHO Expert Consultation on Food Allergen Risk Assessment [19].

Current management relies on strict avoidance and emergency treatment, which does not address the ongoing risk of accidental exposure or the negative impact on quality of life. In this context, immunotherapeutic approaches, particularly oral immunotherapy (OIT), have emerged as potential disease‐modifying treatments for sesame allergy. OIT involves daily ingestion of gradually increasing amounts of sesame protein, typically escalated every 2–4 weeks until a maintenance dose is achieved, which is then continued long term. Sesame OIT requires specialized centers with experienced allergists and entails significant patient commitment, including frequent visits, strict adherence to dosing, and education regarding adverse reaction management. To date, no registered sesame‐specific allergen immunotherapy product is available; therefore, sesame OIT protocols rely primarily on food‐based sources of sesame protein, such as tahini, sesame powder, or sesame flour. This systematic review aims to evaluate the efficacy and safety of sesame immunotherapy in achieving desensitization and improving clinical outcomes in patients with sesame allergy.

## 2 Methods

### 2.1 Search Strategy

A comprehensive literature search was conducted across four electronic databases: PubMed, Embase, Web of Science, and the Cochrane Library The search covered all records available from database inception up to August 2025. Search terms in each database included all relevant subject headings, abstracts, and/or full‐text terms related to IgE‐mediated sesame allergy and allergen‐specific immunotherapy administered via oral, sublingual, epicutaneous, or subcutaneous routes.

The complete databases search strategy is presented in the Supporting Information S1. In addition, the reference lists of all eligible publications were manually reviewed (citation searching) to identify potentially relevant studies not captured through the electronic search.

All records retrieved from the databases were imported into EndNote (Clarivate Analytics, 2025) for reference management. Duplicate citations were removed prior to screening. Three reviewers (J.Z., K.K., A.K.Ł.) independently assessed titles and abstracts for relevance. Articles deemed potentially eligible were subsequently evaluated in full text. Any disagreements or uncertainties regarding study eligibility were resolved through discussion, with arbitration by a fourth reviewer (K.G.) when required.

The review protocol was prospectively registered in the International Prospective Register of Systematic Reviews (PROSPERO; registration number: CRD420251087250). The conduct and reporting of this review adhered to the Preferred Reporting Items for Systematic Reviews and Meta‐Analyses (PRISMA) recommendations.

#### 2.1.1 Eligibility Criteria

Studies were included if they fulfilled all of the following criteria:

- Population: human participants of any age with a confirmed sesame allergy;
- Intervention: sesame allergen immunotherapy administered orally, sublingually, subcutaneously, or epicutaneously;
- Comparator: placebo or absence of a comparator group;
- Outcomes:
  ◦ Primary outcomes:
    1. Desensitization, defined as improvement in OFC outcomes during treatment, including increased tolerated doses;
    2. Sustained unresponsiveness, defined as the ability to consume sesame safely after cessation of therapy.
  ◦ Secondary outcomes:
    1. Health‐related quality of life;
    2. Frequency of adverse events per administered dose;
    3. Use of adrenaline for treatment of anaphylaxis per dose;
    4. Mortality.

Studies were excluded if they were duplicate publications, secondary reports of the same study population, non‐human investigations, conference abstracts, or single‐patient case reports.

### 2.2 Data Extraction and Quality Assessment

For each eligible study, data were independently extracted by two reviewers (J.Z. and K.K.) using a standardized data extraction form. Extracted information included study setting, design, participant characteristics, sample size, intervention and comparator details, reported outcomes, main results, and potential confounding variables. Any discrepancies in data extraction were resolved through discussion, with arbitration by a senior reviewer (K.G.) when consensus could not be reached.

Risk of bias was independently evaluated by two reviewers (J.Z., K.K.) without masking to study authorship or journal. Disagreements were resolved through discussion, with arbitration by a senior reviewer (K.G.) when necessary. The methodological quality of the included studies was assessed using the appropriate Joanna Briggs Institute (JBI) Critical Appraisal Checklist according to study design [20, 21, 22]. For one study‐with cohort‐type characteristics, but a single‐arm, non‐comparative design, the JBI case series checklist was used instead of the cohort checklist, as it was more appropriate for non‐comparative data.

The JBI appraisal tools allow evaluation of domains including randomization, deviations from intended interventions, completeness of outcome data, outcome measurement, and selective reporting. Each domain was rated as indicating low risk of bias, some concerns, or high risk of bias, and an overall risk‐of‐bias judgment was derived based on the domain‐level assessments.

## 3 Results

A total of 100 records were screened by title and abstract for eligibility (Records screened, *n* = 100). Of these, 77 records were excluded at the screening stage, leaving 23 reports sought for retrieval. In addition, one record was identified through citation searching and underwent eligibility assessment. Full‐text assessment resulted in the exclusion of 15 reports due to similar cohort (*n* = 4) or multifood immunotherapy studies without results specific to sesame (*n* = 11). Ultimately, 9 studies meeting the predefined inclusion criteria were included in the systematic review (Tables 1, 2, 3, 4). Except for one study, all used commercially available food products (e.g., tahini, sesame powder, or sesame flour) as the sesame allergen source. The remaining study [30] used a standardized pharmaceutical‐grade dry powder containing proteins from 15 common allergenic foods and was sponsored by a biotechnology company. The study selection process is summarized in the PRISMA flow diagram (Figure 1).

**TABLE 1:** Risk of bias summary table with justification and verdict for studies accessed with the JBI (Joanna Briggs Institute) checklist for case series.

Study ID	JBI1: Clear criteria for inclusion?	JBI2: Condition measured in a reliable way?	JBI3: Valid methods for identification of the condition?	JBI4: Consecutive inclusion?	JBI5: Complete inclusion?	JBI6: Clear reporting of demographics?	JBI7: Clear reporting of clinical information?	JBI8: Outcomes or follow up results clearly reported?	JBI9: Clear reporting of site(s)/clinic(s) demographic information?	JBI10: Statistical analysis appropriate?	Final verdict
You et al. (2022) [6]	YES	YES	YES	UNCLEAR	UNCLEAR	YES	YES	YES	YES	YES	Low to moderate risk of bias
Chua et al. (2024) [23]	YES	YES	YES	UNCLEAR	UNCLEAR	YES	YES	YES	YES	YES	Low to moderate risk of bias
Shah et al. (2025) [24]	YES	UNCLEAR	NO	UNCLEAR	UNCLEAR	YES	YES	NO	YES	YES	High risk of bias

**TABLE 2:** Risk of bias summary table with justification and verdict for studies accessed with the JBI (Joanna Briggs Institute) checklist for quasi‐experimental studies.

Study ID	JBI1: Clear what is “cause” and “effect”?	JBI2: Control group?	JBI3: Participants included in comparisons similar?	JBI4: Participants included in any comparisons receiving similar treatment/care other than exposure or intervention?	JBI5: Measurements of the outcome, both pre and post intervention or exposure?	JBI6: Outcomes of participants included in any comparisons measured in the same way?	JBI7: Outcomes measured in a reliable way?	JBI8: Follow‐up complete and if not, described and analyzed?	JBI9: Statistical analysis appropriate?	Final verdict
Andorf et al. (2017) [25]	YES	NO	N/A	N/A	YES	N/A	YES	YES	YES	High risk of bias
Nachson et al. (2019) [26]	YES	YES	NO	YES	YES	UNCLEAR	YES	YES	YES	
Salari et al. (2022) [27]	YES	NO	N/A	N/A	YES	N/A	YES	YES	YES	

**TABLE 3:** Risk of bias summary table with justification and verdict for studies accessed with the JBI (Joanna Briggs Institute) checklist for RCT studies.

Study ID	JBI1: True randomization?	JBI2: Allocation concealed?	JBI3: Treatment groups similar at baseline?	JBI4: Participants blind to treatment assignment?	JBI5: Those delivering treatment blind to treatment assignment?	JBI6: Treatment groups treated identically?	JBI7: Outcome assessors blind to treatment assignment?	JBI8: Outcomes measured in the same way?	JBI9: Outcomes measured in a reliable way?	JBI10: Follow up complete and if not, described and analyzed?	JBI11: Analyzed in randomized group?	JBI12: Statistical analysis appropriate?	JBI13: Trial design appropriate and any deviations from the standard RCT design?	Final verdict
Andorf et al. (2018) [28]	YES	YES	YES	YES	YES	YES	YES	YES	YES	YES	YES	YES	YES	Low risk of bias
Andorf et al. (2019) [29]	YES	YES	UNCLEAR	YES	YES	YES	YES	YES	YES	YES	YES	YES	YES	Moderate risk of bias
Kim et al. (2024) [30]	YES	YES	UNCLEAR	YES	YES	YES	YES	YES	YES	YES	YES	YES	YES	

**TABLE 4:** Characteristics and outcomes of clinical trials evaluating sesame immunotherapy.

Study ID	Design	Additional intervention (apart from IT)	Inclusion criteria	Number of participants:	Age of the patients (median)	RCT	Comparator	OFC at baseline	Build‐up phase	Starting dose (mg protein)	Maintenance dose (mg)/duration of maintenance period (mo)	Discontinued	End points	Results	Adverse reactions
Nachshon et al. Israel, 2019 [26]	Prospective, open‐label, single‐center study	No	Positive OFC, or A clinical history of an immediate reaction (within 2 hours of exposure) to sesame following accidental ingestion in the past year and/or systemic symptoms, together with evidence of sesame sensitization, defined as a positive SPT and/or sesame‐specific serum IgE ≥ 0.35 kUA/L	Immunotherapy group (*n* = 60); sesame avoidance control group (*n* = 15)	5.8–11.6 (7.5) years old	No	Standard care (dietary avoidance)	Yes	6.5 (3.8–12.8) months	Based on ED	1200/≥ 6 (median 8)	4 patients	OFC tolerance 4000 mg sesame protein	Fifty‐three OIT‐treated patients (88.3%) were fully desensitized to sesame, compared with 0% of controls	Reactions occurred in 4.7% of hospital‐administered doses and 1.9% of home‐administered doses. Epinephrine‐treated reactions occurred in 16.7% of patients during hospital dosing and 8.3% during home dosing.
Salari et al. Iran, 2022 [27]	Prospective, open‐label, single‐center study	Omalizumab 150 mg	Positive oral food challenge (OFC) in patients with a history of anaphylaxis. With or without positive allergy tests (SPT, SPP, or sesame‐specific IgE).	11 patients	25–55 (41) years old	No	None (single‐arm study)	Yes	ND	0.003	5000/4	0	OFC tolerance 5000 mg sesame protein	All 11 patients achieved desensitization.	Skin reactions (itching, urticaria, flushing, erythema, angioedema): 100% Cardiovascular reactions (tachycardia, hypertension, hypotension): 45.5%. Gastrointestinal reactions (abdominal cramps, vomiting): 36.4%. Respiratory reactions (cough, wheezing, dyspnea): 27.3%. No patients required epinephrine treatment.
Chua et al. Canada, 2024 [23]	Prospective, open‐label, single‐center study	No	Preschoolers with a history of objective reaction to sesame. Either a positive SPT (wheal ≥ 3 mm) or sesame‐specific IgE ≥ 0.35 kU/L.	28 patients	23.3–41.8 (33) months old	No	None	In some patients	ND	3.0	200/12	2 patients	OFC tolerance ≥ 2000 mg sesame protein	Exit OFC was performed in 91.3% of eligible subjects, with 85.7% (*n* = 18/21) completing it successfully. During OFC, 4.8% had grade 1% and 9.5% had grade 2 reactions.	During the buildup phase, 32.1% had no reactions, 28.6% had grade 1 reactions, and 39.3% had grade 2 reactions. 3.57% required epinephrine for a grade 2 reaction.
You et al. USA, 2022 [6]	Retrospective study	No	Convincing history of an allergic reaction with a positive sesame SPT or Positive OFC	23 patients	1.19–18.51 (8.5 ± 4.7) years old	No	None (single‐arm study)	In some patients	293.7 ± 87.1 days	0.1	600/≥ 3	3 patients	Maintenance achievement; safety/QoL	Food‐related anxiety (−0.773) and social/dietary limitation (−0.687) showed significant improvement in quality of life.	During the buildup phase, gastrointestinal symptoms occurred in 91.3%, cutaneous symptoms in 78.3%, and respiratory reactions in 26% of patients. Systemic reactions were rare (0.513% of adverse reactions), occurred in 1 patient and required a single dose of epinephrine. During the buildup phase, adverse reactions occurred in 6.88% of clinic doses and 2.47% of home doses. 2 systemic reactions (12.5%) occurred during maintenance phase and required epinephrine.
Shah et al. USA, 2025 [24]	Retrospective review	No	Convincing history of an IgE‐mediated reaction to sesame with either a positive SPT or sesame‐specific IgE ≥ 0.35 kUA/L, or Diagnosis of sesame allergy made by the primary allergist, or Failure to tolerate tahini during OFC	84 patients	0.83–11(2) years old	No	None	In some patients	ND	6.0	833/> 6	16 patients during buildup 2 patients during maintenance	SU after sesame OIT (2500 mg sesame protein OFC)	97% (*n* = 30/31) tolerated the desensitization OFC. 97% (*n* = 29/30) underwent SU OFC and all tolerated 2500 mg after ≥ 4 weeks of avoidance. 2 patients who paused maintenance dosing tolerated direct SU OFC.	Allergic reactions occurred in 25 patients during the OIT course. 96% of them experienced grade 1 reactions. The most common reactions were localized erythema and/or urticaria, followed by abdominal pain and/or limited emesis. 2 patients required epinephrine—both patients subsequently achieved maintenance.
Andorf et al. USA, 2018 [28]	Randomized, double‐blind controlled Phase 2 trial	Multifood IT, omalizumab	Age 4–55 years (enrolled: 5–22 years). Multiple food allergies confirmed by DBPCFC within 12 months (reaction at ≤ 125 mg; cumulative 500 mg protein). Positive skin prick test (≥ 6 mm) and/or food‐specific IgE > 4 kU/L for each food. Total IgE < 2000 kU/L. Reached ≥ 1 g maintenance dose per food by weeks 28–29 to enter randomized phase. * The full inclusion criteria are detailed in the study protocol and are not presented in the manuscript.	70 enrolled, 60 randomized patients (including 8 with sesame allergy)	5–22 years old (mean 10.8 years; median not reported)	Yes	Discontinued dosing after desensitization	Yes, in the Phase 1	29 weeks	—	≥ 1000 (of each food allergen in the multi‐OIT)/1.5	7 patients discontinued following randomization	OFC tolerance 2000 mg of at least one food	The primary endpoint was tolerance of 2 g of ≥ 2 allergens. 85% of participants on active OIT achieved the primary endpoint compared with 55% in the discontinuation arm. Success rates increased with a higher number of allergens in the active OIT group, reaching 100% in participants with 4 or 5 foods. SESAME: Among patients undergoing IT, one received a 1 g dose, four received 300 mg, and three were assigned to the 0 mg (discontinuation) group. At week 36, only one patient in the 0 mg group failed the OFC.	No clinically significant differences in adverse event rates across study arms. Antihistamine and inhaler use were similar between all treatment groups. Epinephrine use was infrequent and occurred more often in the 1 g arm (4 doses) than in the discontinuation (1 dose) or 300 mg (0 doses) arms.
Andorf et al. USA, 2017 [25]	Observational study	Multifood IT	Patients reached a 2 g maintenance dose per food during the initial Phase 1 trial.	46 patients (including 5 with sesame allergy)	6.4–46.9 (10,6) years old	No	Low‐dose maintenance group (vs. high‐dose maintenance group)	Yes	—	—	300 or 2000/48 (27–72)	1 patient	SU after OIT (2 g protein)	100% of patients tolerated 2 g of each food allergen protein during OFCs performed at the end of the study.	1207 reactions occurred (2.29% of maintenance doses). 88.9% were mild and 10.69% were moderate. 0.41% were severe, limited to nasal and skin symptoms. No fatal or serious adverse events occurred. No reactions required epinephrine.
Kim et al. USA, 2025 [30]	Multi‐center, Phase 1/2 randomized, double‐blind, placebo‐controlled trial	Multifood IT	4–55 years old; the primary analysis focused on children aged 4–17 years. Participants had 1–5 qualifying food allergies, defined by dose‐limiting symptoms at ≤ 100 mg during screening DBPCFC. Qualifying allergens included almond, cashew, egg, fish, milk, tree nuts, peanut, sesame, shellfish, soy, wheat, and salmon.	61 randomized pediatric patients (including 3 with sesame allergy)	4–17 years old (10‐median age at screening)	Yes	Active comparator (low‐dose ADP101) and placebo control	Yes	38 weeks	5	1500 mg/daily; 100 mg protein per food or 4500 mg/daily; 300 mg Protein per food or placebo (low‐dose or high‐dose placebo)/10	14 pediatric patients	DBPCFC tolerance ≥ 600 mg protein of a single qualifying food	The proportion of pediatric participants tolerating ≥ 600 mg of at least one qualifying food was 20.0% in the placebo group, 38.1% in the low‐dose ADP101 group and 55.0% in the high‐dose ADP101 group Sesame: Three patients had a sesame allergy; two underwent OIT with a low dose and one with a high dose ADP101, and all resulted in failed desensitization.	During updosing, AESIs occurred in 15.0% (placebo), 23.8% (LD‐ADP101), and 35.0% (HD‐ADP101) of participants. Anaphylaxis during updosing occurred in 15.0%, 19.0%, and 30.0% of placebo, low‐dose, and high‐dose groups, respectively. Epinephrine use during updosing occurred in 5.0% (placebo), 14.3% (low‐dose), and 35.0% (high‐dose) participants. Grade ≥ 3 events during updosing occurred in 5.0% of placebo and 5.0% of high‐dose participants; 0% in low‐dose. During maintenance, AESIs occurred in 5.0% (placebo), 0% (low‐dose), and 5.0% (high‐dose) participants, with similar rates for anaphylaxis and epinephrine use. One eosinophilic esophagitis event occurred in the low‐dose group (4.8%).
Andorf et al. USA, 2019 [29]	Randomized, double‐blind, placebo‐controlled clinical trial	Multifood IT, omalizumab	Positive DBPCFC at ≤ 500 mg of food protein Positive SPT wheal ≥ 6 mm above the negative control) and/or Food‐specific IgE > 4 kU/L for each food	48 patients (including 8 with sesame allergy)	4–15 years (8 years in the omalizu‐mab group and 7 years in the placebo group)	Yes	Placebo	Yes	Median time: 18 weeks in the omalizumab arm; 24 weeks in the placebo arm.	5–1250 mg per food (based on the IDED)	Up to 2 g of each food.	Not specified	Desensitization 2 g protein/allergen for any 2 foods	At week 36, 83% of omalizumab versus. 33% of placebo participants passed DBPCFCs to ≥ 2 foods Sesame: All 8 participants with sesame allergy passed the week‐30 OFC, indicating desensitization. Participants were subsequently assigned to 1 g (*n* = 1), 300 mg (*n* = 4), or 0 mg (*n* = 3) groups; at week 36, only one participant in the 0 mg group failed the final OFC.	No serious adverse events or severe (grade 3) adverse events were reported. Weeks 8–16: Omalizumab was associated with fewer AE‐related IT doses compared with placebo.

Abbreviations: AESI = adverse events of special interest; DBPCFC = double‐blind, placebo‐controlled food challenge; IDED = initial dose escalation day; IT = immunotherapy; ND = not disclosed; OFC = oral food challenge; RCT = randomized controlled trial; SPT = skin prick test, SU = sustained unresponsiveness.

**FIGURE 1:**
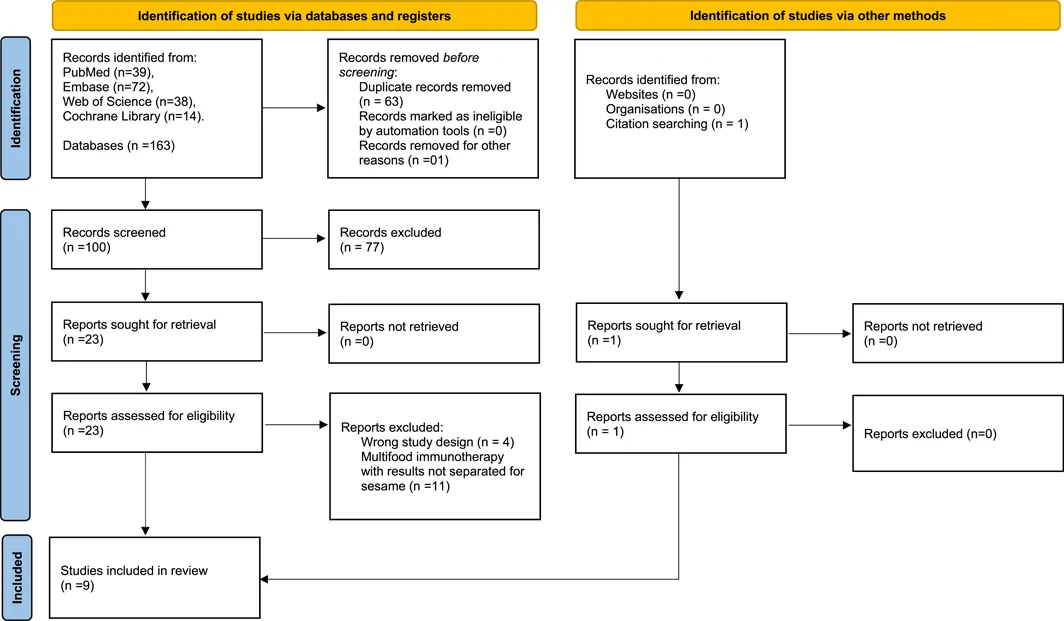
PRISMA flow diagram.

### 3.1 Effectiveness and Safety of Sesame Oral Immunotherapy

#### 3.1.1 Single‐Allergen Sesame OIT

Four studies [23, 24, 26, 27] evaluated OIT targeting sesame alone, including two quasi‐experimental studies and two cohort or case series studies. Study protocols differed substantially with respect to participant age (ranging from infancy to adulthood), build‐up schedules, maintenance doses (200–5000 mg sesame protein), duration of maintenance (4 months to ≥ 1 year), and outcome definitions (final OFCs ranging from 2000 to 5000 mg).

Nachshon et al. [26] evaluated 60 patients aged ≥ 4 years with sesame allergy confirmed by positive OFC, along with 15 controls diagnosed by OFC or a convincing clinical history supported by positive allergy testing. Following the build‐up phase, 53 patients (88.3%) achieved desensitization to 4000 mg of sesame protein and continued maintenance at 1200 mg. Forty‐six patients underwent a final OFC after at least 6 months of maintenance, all of whom successfully tolerated 4000 mg. No spontaneous tolerance was observed in the control group. Epinephrine was administered in 10 patients (16.7%) during clinic visits and in 5 patients (8.3%) during home dosing. Adverse reactions occurred during 4.7% of induction doses and 2% of home doses.

Chua et al. [23] investigated low‐dose sesame OIT (200 mg sesame protein) in preschool‐aged children (9–70 months) with a history of objective reactions and positive allergy testing. Of 28 enrolled patients, 21 completed the final OFC (to a maximum dose of 2000 mg) after one year of maintenance, with 18 (85.7%) achieving a negative challenge. One patient required epinephrine. Most reactions were mild to moderate (grade 1–2).

Shah et al. [24] reported outcomes of sesame OIT in a pediatric cohort (median age 2 years; range 0.83–11 years) using a maintenance dose of approximately 1000 mg of sesame protein. Eighty‐four patients initiated OIT, of whom 65 reached the maintenance phase. After at least six months of maintenance dosing (median duration: 17 months), patients demonstrating a decrease in SPT wheal size and/or sesame‐sIgE levels were offered a final desensitization OFC with 2500 mg of sesame protein. Of the 31 patients who underwent this challenge, 30 (96.7%) successfully passed. Sustained unresponsiveness was evaluated in a subset of participants after a 4–6‐week period of complete dosing cessation; 29 of 30 patients (96.7%) passed a repeat OFC following this interruption. Two additional patients who discontinued daily dosing due to intercurrent illness or adherence difficulties subsequently passed OFCs. Epinephrine was administered in two cases, one during home dosing and one during in‐clinic administration.

Salari et al. [27] evaluated sesame OIT combined with adjunctive omalizumab therapy in 11 adults (aged 25–55 years) with a history of sesame‐induced anaphylaxis. Omalizumab was administered at a dose of 150 mg 1 month prior to OIT initiation and again on the day of immunotherapy commencement. The maintenance dose of sesame protein was 5000 mg and was continued for four months. All patients tolerated 5000 mg of sesame protein during the final OFC with no epinephrine use reported.

#### 3.1.2 Multifood Oral Immunotherapy Including Sesame

Data on sesame immunotherapy administered concurrently with other food allergens (multifood OIT) remain limited. In this review, only four studies [25, 28, 29, 30] were included in which sesame‐specific outcomes could be identified or extracted. Collectively, these studies suggest that desensitization to sesame may be achievable when administered in parallel with OIT for other allergens; however, conclusions are constrained by small sample sizes and limited sesame‐specific reporting.

Kim et al. [30] evaluated multifood OIT involving 15 allergens, including sesame. Participants received either a low dose (100 mg protein per allergen) or a high dose (300 mg protein per allergen), compared with placebo. Successful outcome was defined as a negative OFC with a threshold ≥ 600 mg for at least one allergen. Efficacy rates were 55% in the high‐dose group, 38.1% in the low‐dose group, and 20.0% in the placebo group. Among pediatric participants with sesame allergy, two in the low‐dose group and one in the high‐dose group failed the final OFCs, indicating persistent allergic reactivity, whereas one adult participant with sesame allergy did not complete the study.

In a single‐center Phase 1 study by Andorf et al. [25], the efficacy of multi‐allergen OIT was assessed with long‐term follow‐up. Participants were desensitized to up to five food allergens and followed for up to 72 months after reaching a 2 g maintenance dose. Among five patients with sesame allergy, three received a high maintenance dose (2000 mg) and two a reduced dose (300 mg). All passed final OFCs to 2 g sesame protein, regardless of maintenance dose.

In a randomized, double‐blind pilot study, Andorf et al. [28] subsequently evaluated multifood OIT administered in combination with omalizumab or placebo, with efficacy assessed by end‐of‐study double‐blind, placebo‐controlled food challenges (DBPCFCs) up to 2 g of food protein, with escalation evaluated to 4 g. Eight participants with confirmed sesame allergy were included; a negative final DBPCFC was observed in three subjects receiving omalizumab and in two subjects receiving placebo, while treatment failure occurred in two subjects in the omalizumab group and in one subject in the placebo group.

Andorf et al. [30] later assessed the efficacy of rapid desensitization using omalizumab in individuals with multiple food allergies. All participants received omalizumab from weeks 1–16 and subsequently underwent multi–oral immunotherapy (multi‐OIT) involving 2 to 5 food allergens between weeks 8 and 30. Participants who successfully tolerated a maintenance dose of 1 g of protein for each allergen by weeks 28–29, were randomized in a 1:1:1 ratio to receive 1 g, 300 mg, or 0 mg of each allergen during a double‐blind maintenance phase lasting until week 36. At the end of this phase, OFC to a minimum dose of 2 g of protein per allergen were performed.

Eight participants had sesame allergy and all of them passed the OFC at week 30, consistent with successful desensitization. Participants were subsequently assigned to the 1 g (*n* = 1), 300 mg (*n* = 4), or 0 mg (discontinuation; *n* = 3) groups. At week 36, only one participant in the 0 mg group failed the final OFC. In this subgroup analysis, desensitization appeared to be more consistently maintained with continued maintenance OIT dosing (300 mg or 1 g) compared with complete discontinuation. However, these findings should be interpreted with caution given the limited sample size.

#### 3.1.3 Quality of Life

One study [6] evaluated quality of life outcomes in patients undergoing sesame OIT using the Food Allergy Quality of Life Questionnaire (FAQLQ). Of 28 patients initiating OIT, 21 reached maintenance and 11 completed both pre‐ and post‐treatment questionnaires. Mean age was 8.5 years. Adverse reactions were infrequent, with one systemic reaction during build‐up and two during maintenance requiring epinephrine. Significant improvements were observed in food‐related anxiety and social/dietary limitation domains, indicating meaningful psychosocial benefit.

Although not sesame‐specific, additional studies [31, 32] in mixed‐allergen OIT cohorts consistently demonstrate improvements in emotional well‐being, reduced anxiety related to accidental exposure, and enhanced dietary freedom across pediatric and adult populations, suggesting potential quality‐of‐life benefits applicable to sesame OIT.

#### 3.1.4 Additional Immunotherapy Modalities: Sublingual Immunotherapy

Although evidence for sesame sublingual immunotherapy (SLIT) is limited, emerging data suggest feasibility and safety of real‐food–based SLIT protocols including sesame. Studies by Windom et al. [33] and Williams et al. [34] demonstrated high adherence, absence of systemic reactions requiring epinephrine, and favorable patient satisfaction in multifood SLIT protocols. While sesame‐specific outcomes and efficacy were not assessed, these findings suggest that SLIT may represent a potential alternative therapeutic option for selected patients.

#### 3.1.5 Safety Considerations

No deaths related to sesame immunotherapy were reported in any of the included studies. Across studies, most adverse events associated with sesame OIT were mild and consistent with the safety profile reported for multifood OIT protocols. However, severe reactions were also documented. In the cohort reported by Nguyen et al. [35], one patient experienced a severe reaction to sesame during the initial dose escalation procedure and required three doses of epinephrine followed by transfer to the pediatric intensive care unit.

In addition to the studies formally included in this review, several publications reporting safety outcomes in mixed‐allergen OIT cohorts, largely overlapping with the primary sesame cohorts, were reviewed narratively. These analyses focused on specific safety outcomes, including epinephrine‐treated reactions during home dosing, severe anaphylactic reactions, and identifiable reaction triggers. Sesame‐specific data were extracted when available; however, because risk factor analyses were not performed on an allergen‐specific basis, these findings are presented as supportive safety data rather than as direct evidence specific to sesame OIT.

In analyses by Nachshon et al. [36], a total of 757 OIT treatments were administered to 644 patients with peanut, tree nut, sesame, and egg allergies. Home epinephrine‐treated reactions (HETRs) occurred in 83 patients (10.9%). Among 115 patients undergoing sesame OIT, 7 patients (6.1%) experienced HETRs. Risk factor analyses conducted across all allergens demonstrated that most reactions occurred at doses below 300 mg of protein. Identified cofactors included physical exercise (19%), fatigue (12%), and intercurrent illness (10%), while no identifiable trigger was reported in 34% of reactions. Across all allergens, prior emergency department–treated reactions and epinephrine use during in‐clinic induction were associated with an increased risk of HETRs. Asthma was not associated with an increased risk, whereas older age and a lower single highest tolerated dose (SHTD) at OIT initiation were associated with higher HETR rates. When compared with other allergens, sesame OIT was associated with a lower risk of HETRs than tree nut OIT (adjusted hazard ratio 0.28, *p* = 0.003).

In a subsequent analysis [37] including 1637 OIT treatments, the same 115 patients undergoing sesame OIT were analyzed. Thirty high‐grade home epinephrine‐treated reactions (HG‐HETRs) were reported, representing 1.65% of all OIT treatments. Only one HG‐HETR occurred among patients undergoing sesame OIT, corresponding to 0.87% of sesame treatments.

## 4 Discussion

This systematic review summarizes the current evidence on sesame OIT within the broader context of emerging immunotherapeutic approaches for food allergies. A quantitative meta‐analysis was not feasible because the available evidence is derived predominantly from small, heterogeneous case series and quasi‐experimental studies with substantial variability in design, populations, and outcome measures. While an ideal evaluation of sesame OIT would involve randomized, placebo‐controlled trials with standardized diagnostic and outcome assessments, practical and methodological challenges—including difficulties with blinding and the demanding nature of OIT protocols—likely contribute to the current lack of high‐quality data. Consequently, the findings of this review should be interpreted with caution.

Across single‐allergen sesame OIT studies, desensitization rates were consistently high, ranging from approximately 85% to over 95%, despite heterogeneity in maintenance doses (200–5000 mg of sesame protein), participant age, and study design. These findings are comparable with OIT outcomes reported for peanut, cow's milk, and hen's egg allergies, where systematic reviews of randomized controlled trials have demonstrated that daily oral exposure can significantly increase the threshold of reactivity and reduce the risk of accidental reactions [38, 39]. Such parallels support the notion that OIT represents a broadly applicable modality for food allergens, including less‐studied allergens like sesame.

Sustained unresponsiveness remains an important endpoint in immunotherapy. The pediatric cohort reported by Shah et al. demonstrated high rates of sustained tolerance after a 4–6‐week period of dosing cessation, which aligns with observations in other food allergies where OIT may induce transient or longer‐lasting tolerance in a subset of patients. However, as with other allergens, long‐term immune tolerance in sesame allergy is not yet well characterized, highlighting the need for continued follow‐up and standardized evaluation protocols.

Adjunctive use of omalizumab appears to improve both safety and efficacy of OIT in high‐risk individuals, such as adults with a history of anaphylaxis to sesame. This strategy mirrors findings from peanut and multifood OIT trials, where short‐term biologic therapy facilitated rapid dose escalation and improved tolerability [40]. While promising, small sample sizes and short‐term follow‐up preclude definitive conclusions regarding sustained benefit and cost‐effectiveness.

Safety considerations remain central to the clinical application of sesame OIT. Most adverse events are mild, consistent with the profile reported for other food allergens but severe reactions can occur, particularly during initial dose escalation.

Quality‐of‐life improvements observed in sesame OIT participants, including reduced food‐related anxiety and enhanced social and dietary freedom, parallel findings from OIT studies for other major allergens, reinforcing the psychosocial benefits of desensitization beyond strictly immunologic endpoints [41].

Emerging approaches, such as SLIT using real‐food preparations, show feasibility and safety in multifood protocols, although sesame‐specific efficacy remains to be established. These alternative modalities reflect a broader trend in food allergy immunotherapy toward personalized strategies that balance efficacy, safety, and patient preference.

In line with EAACI guidelines [42], allergen‐specific immunotherapy is recommended for peanut allergy in children and adolescents, while milk and egg OIT are conditionally suggested; however, no formal recommendation currently exists for sesame immunotherapy. Accordingly, sesame OIT should be considered a specialized or investigational intervention, reserved for carefully selected patients.

In summary, sesame OIT demonstrates efficacy and an acceptable safety profile comparable to that reported for other common food allergens. While current evidence is limited by small sample sizes, heterogeneity in protocols, and short follow‐up, these findings support the potential role of sesame OIT in specialized clinical settings. Future research should focus on randomized trials, standardized outcome measures, long‐term assessment of sustained unresponsiveness, and quality‐of‐life endpoints, with consideration of adjunctive biologics and alternative delivery modalities.

## 5 Conclusions

Sesame OIT is a promising strategy for managing sesame allergy, achieving clinically meaningful desensitization in both children and adults with an overall favorable safety profile. While severe reactions are uncommon, careful administration in specialized settings is required, particularly during dose escalation. Adjunctive use of omalizumab may improve safety and enable faster up‐dosing in high‐risk patients. Sesame OIT is associated with improved quality of life, including reduced anxiety and increased dietary flexibility. Although sustained unresponsiveness is achievable in many patients, long‐term immune tolerance remains insufficiently defined. Alternative immunotherapeutic approaches, including SLIT, remain investigational with limited sesame‐specific evidence. Further well‐designed studies are needed to establish optimal protocols, long‐term outcomes, and patient‐centered benefits.

## Author Contributions

**Joanna Zielińska:** conceptualization, writing – original draft, investigation, data curation. **Katarzyna Kunkiel:** methodology, writing – review, conceptualization, investigation, data curation, visualization. **Anna Krupa‐Łaska:** investigation, data curation, visualization. **Marek Kulus:** supervision, writing – review and editing. **Katarzyna Grzela:** supervision, writing – review and editing. All authors meet the criteria for authorship as defined by the journal. All authors have read and approved the final manuscript and agree to be accountable for all aspects of the work.

## Funding

The authors have nothing to report.

## Ethics Statement

This study is a systematic review conducted in accordance with the PRISMA 2020 reporting guidelines. The review was based exclusively on data from previously published studies and publicly available sources. Therefore, ethical approval and informed consent were not required for this study. The conduct of this review complied with the principles of the Declaration of Helsinki where applicable.

## Conflicts of Interest

The authors declare no conflicts of interest.

## Supporting information


Supporting Information S1

## Data Availability

All data analyzed in this study were obtained from previously published articles cited in the reference list. No new datasets were generated. The extracted data supporting the findings of this review are available from the corresponding author upon reasonable request.
