# Supplementary material for: Sesame Immunotherapy: A Systematic Review and Narrative Synthesis
Source: Clin Transl Allergy. 2026 Sep 29;16(10):e70206. doi: 10.1002/clt2.70206 (PMC13621480; doi:10.1002/clt2.70206)
Supplement: Supplementary file 1 [file CLT2-16-e70206-s001.docx]

Supplementary Material A

Pubmed search strategy

((("sesamum"[MeSH Terms] OR "sesamum"[All Fields] OR "sesame"[All Fields]) AND ("immunotherapy"[MeSH Terms] OR "immunotherapy"[All Fields] OR "immunotherapies"[All Fields] OR "immunotherapy s"[All Fields] OR ("desensitise"[All Fields] OR "desensitised"[All Fields] OR "desensitiser"[All Fields] OR "desensitises"[All Fields] OR "desensitising"[All Fields] OR "desensitization, immunologic"[MeSH Terms] OR ("desensitization"[All Fields] AND "immunologic"[All Fields]) OR "immunologic desensitization"[All Fields] OR "desensitisation"[All Fields] OR "desensitization"[All Fields] OR "desensitizations"[All Fields] OR "desensitize"[All Fields] OR "desensitized"[All Fields] OR "desensitizer"[All Fields] OR "desensitizers"[All Fields] OR "desensitizes"[All Fields] OR "desensitizing"[All Fields]))) NOT ("case reports"[Publication Type] OR "editorial"[Publication Type] OR "letter"[Publication Type] OR "review"[Publication Type])) NOT ("animals"[MeSH Terms] NOT "humans"[MeSH Terms])

Embase search strategy

((('sesame'/exp OR 'sesame') OR ('sesamum'/exp OR 'sesamum')) AND (('immunotherapy'/exp OR 'immunotherapy') OR ('desensitization'/exp OR 'desensitization') OR ('anti anaphylaxis'/exp OR 'anti anaphylaxis') OR ('antianaphylaxis'/exp OR antianaphylaxis))) AND 'human'/de AND ('article'/it OR 'article in press'/it OR 'conference paper'/it OR 'clinical trial'/it)

Web of Science search strategy

(TS=(sesame or sesamum) AND TS=(Desensitization or Desensitisation or immunotherapy)) NOT (DT==("REVIEW" OR "MEETING ABSTRACT" OR "EDITORIAL MATERIAL" OR "LETTER"))

Cochrane search strategy

<https://www-1cochranelibrary-1com-1000005je1229.han3.wum.edu.pl/advanced-search/search-manager?search=7756085>
